# Establishment of an animal model of vascular dementia

**DOI:** 10.3892/etm.2014.1926

**Published:** 2014-08-21

**Authors:** HAO WANG

**Affiliations:** Department of Traditional Chinese Internal Medicine, China-Japan Friendship Hospital, Beijing 100029, P.R. China

**Keywords:** vascular dementia, animal model, mouse

## Abstract

The aim of this study was to establish a mouse model of vascular dementia (VaD) in order to overcome the shortcomings of rat models of VaD, which include high production costs, difficult surgery, surgical trauma and high mortality. In this study, repeated ischemia-reperfusion of the total bilateral carotid artery in mice, combined with a reduction of blood pressure, was used to establish an animal model of VaD. A total of 40 Kunming mice (clean grade) were randomly divided into a sham group and a model group. Behavioral tests were performed for each group following the surgery, and the morphology of the hippocampus was analyzed. The results of the step-down avoidance test, water maze test and microscopy examinations confirmed that following surgery, learning and memory dysfunction was significantly increased in the model group. The results of the morphological observations showed that the number of hippocampal CA1 neurons was significantly decreased in the model group compared with that in the sham surgery group (P<0.01). In the present study, a mouse model of VaD was successfully established, which was simple, effective and reliable, and may be used in the future to investigate VaD.

## Introduction

Elderly individuals are the fastest growing segment of the population in Western countries. Over half of elderly individuals have dementia; however, the etiology of dementia has yet to be elucidated (1). A number of causes of dementia have been identified, and vascular risk factors (VRF) are associated with a higher incidence of dementia (2). Patients with vascular dementia (VaD) are often isolated and withdrawn from society due to negative symptoms and functional disabilities (3), which are characterized by functionally impairing deterioration of memory, language, personality or visuospatial skills (4).

The pathogenesis of dementia remains unclear and effective drugs for the treatment of this disease are lacking. Therefore, the development of animal models of VaD is important for use in studies to understand the pathophysiology of dementia, as well as to determine the efficacy of novel therapeutic drugs. Currently, rats are usually used as VaD animal models; however, establishing rat models of VaD is expensive and challenging. Therefore, in the present study, repeated ischemia-reperfusion (IR) of the total bilateral carotid artery, combined with blood pressure reduction, was used to establish a VaD model in mice. The mice were subjected to behavioral tests to identify whether they had significant postoperative learning and memory dysfunction, impaired spatial orientation constancy and an impaired spatial recognition ability. In addition, the number of hippocampal CA1 neurons was investigated. The suitability of this animal model of VaD for use in the study of the pathogenesis and prevention of VaD was thus assessed.

## Materials and methods

### Animals

A total of 40 Kunming mice (clean grade; 20 male and 20 female), weighing between 20 and 22 g, were provided by the Experimental Animal Center of the Chinese Academy of Medical Sciences (Beijing, China). The present study was performed in accordance with the recommendations in the Guide for the Care and Use of Laboratory Animals (National Institutes of Health, 8th Edition, Bethesda, MD, USA). The animal protocol was reviewed and approved by the Institutional Animal Care and Use Committee (IACUC) at the China-Japan Friendship Hospital (Beijing, China).

### Establishment of the animal model

The mice were anesthetized via intraperitoneal injection with 480 mg/kg 10% chloral hydrate and then the anterior cervical skin was disinfected with 75% alcohol. An anterior neck middle incision was made, and the bilateral carotid arteries were separated, and then a thread was passed below each carotid artery for closure. Sodium nitroprusside (Beijing Double-Crane Pharmaceutical Co., Ltd., Beijing, China) was intraperitoneally injected (3.5 mg/kg) to cause hypotension. The bilateral carotid arteries were closed by ligation for 10 min, and then loosened for 10 min, and this was repeated 3 times. The threading was then withdrawn and the incision sutured. The mice were placed in cages for rearing.

### Grouping

The mice were randomly divided into two groups: a sham group and a model group (each with 10 mice in a 10 days group and 10 mice in a 30 days group. The mice in the sham group were subjected only to peeling of the bilateral common carotid arteries; the blood vessels were not closed and the blood pressure was not lowered. The mice in the model group were subjected to the aforementioned modeling method.

### Step-down avoidance test

A passive avoidance reaction tank, 60×12×33 cm in size (Institute of Materia Medica, Chinese Academy of Medical Sciences, Beijing, China) was used as the jumping test device. It was separated into two equal parts by a black plastic sheet, and an energized copper grid was placed on the bottom. An insulated rubber mat with diameter and height of 4.5 cm was placed on the right of the device, which was used as the safe platform for the mouse to avoid an electric shock. A voltage regulator was used to regulate the voltage.

The mice were placed in the jumping device to adapt for 3 min and then a 40 v alternating current was turned on to shock the mice. The mice jumped to the safe platform to avoid the electric shock. The time from shock occurrence to jumping was record as the response period. The frequency of electric shock (number of errors) and the electric shock time within 5 min were also observed. The response period, number of errors and electric shock time were considered as their learning performance. After 24 h, the test was repeated. The mice were placed on the safe platform and an electric current was applied. The time of mice jumping from the safe platform to the copper grid within 5 min was observed as the incubation period, and the number of electric shocks was taken as the number of errors. The incubation period, number of errors and the electric shock time were considered as the memory performance.

### Water maze test

The water maze was provided by the Institute of Materia Medica, Chinese Academy of Medical Sciences, and was made from black plastic sheeting (water depth, 10 cm; water temperature, 23±1°C). It was divided into four zones, specifically A, B and C zones and an end zone that contained a footboard for the mice to climb out of water. The swim from the A, B and C zones to the end zone was used as a phase of training, and was repeated three times. Subsequently, the time for mice to swim from zone A to the footboard of the end zone (entire swim time) and the frequency of entering the blind side (number of errors) within 5 min were recorded and taken as the learning performance. This was repeated after 24 h, and the data obtained was considered as the memory performance. If the mice did not swim to the end within 5 min, 5 min was used as the whole swim time.

### Morphological observations

After 10 and 30 days following the surgery, mice in each group were selected and were decapitated after anesthesia. The brain was quickly removed, followed by coronal incision. The hippocampus was obtained and was fixed in 20% formalin. Following dehydration with ethanol and embedding with paraffin, 5 μm sections were made, followed by hematoxylin and eosin staining. The hippocampal CA1 region was then observed under an optical microscope (CKX41, Olympus, Tokyo, Japan).

### Statistical analysis

Data are expressed as the mean ± standard deviation. Statistical analysis was performed using SPSS statistical software, version 13.0 (SPSS, Inc., Chicago, IL, USA). A t-test was used to analyze the differences between different groups, and P<0.05 was considered to indicate a statistically significant difference.

## Results

### Behavioral experiments

The response period of the model group 10 days following surgery was longer than that of the sham group, and was markedly more prolonged 30 days following surgery (P<0.001). The incubation period 10 days and 30 days following surgery was significantly shorter than that in the sham group (P<0.01). The number of errors and electric shock time 10 days and 30 days following surgery in the learning and memory periods were significantly increased compared with those in the sham group (P<0.001). This indicates that the learning and memory function of the mice in model group was damaged, and this damage increased with time following the surgery (Tables I and II).

### Water maze test

The learning time and number of errors in the learning and memory periods in the model group 10 days following surgery were significantly increased compared with those in the sham group (P<0.01), and the increases were more pronounced 30 days following surgery (P<0.001). A comparison of the results for the model group 10 days and 30 days following surgery showed that the number of errors in the learning period, and the entire swim time and number of errors in the memory period increased markedly at 30 days (P<0.05), and the entire swim time in the learning period was significantly higher 30 days after surgery (P<0.01). This indicates that the mice in the model group developed a severe spatial resolution disorder, and the extent of the disorder increased with time following the surgery (Tables III and IV).

### Morphological observations

**Figure 1:**
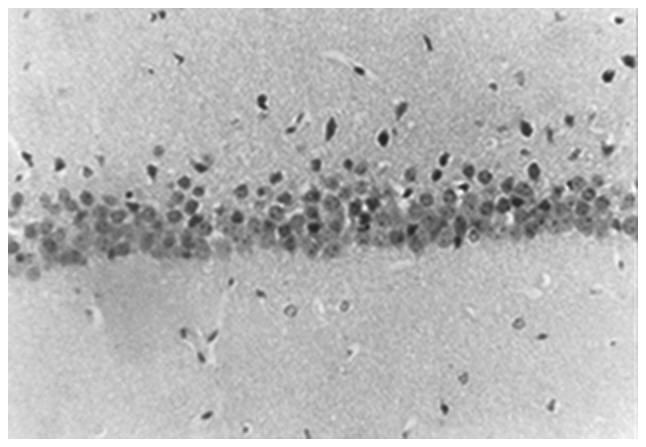
Sham surgery 10 day group: large numbers of nerve cells with closely arranged, neat, rounded nuclei, prominent nucleoli and chromatin uniform (hematoxylin and eosin staining; Magnification, ×50.).

**Figure 2:**
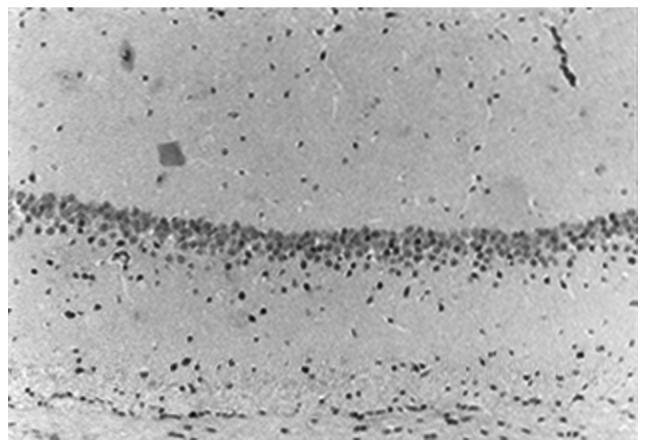
Sham surgery 30 day group: large numbers of nerve cells with closely arranged, neat, rounded nuclei, prominent nucleoli and chromatin uniform (hematoxylin and eosin staining; Magnification, ×25).

**Figure 3:**
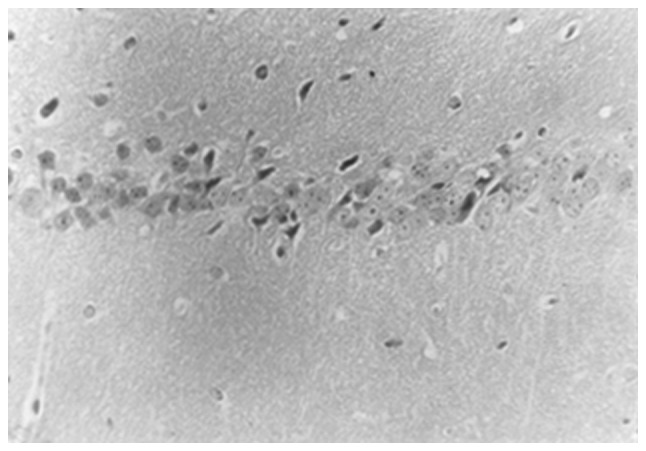
Model 10 day group: reduced number of nerve cells, disorganized, heavily stained, pyknotic nerve nuclei and large irregularly shaped glial cells (hematoxylin and eosin staining;Magnification, ×50.).

**Figure 4:**
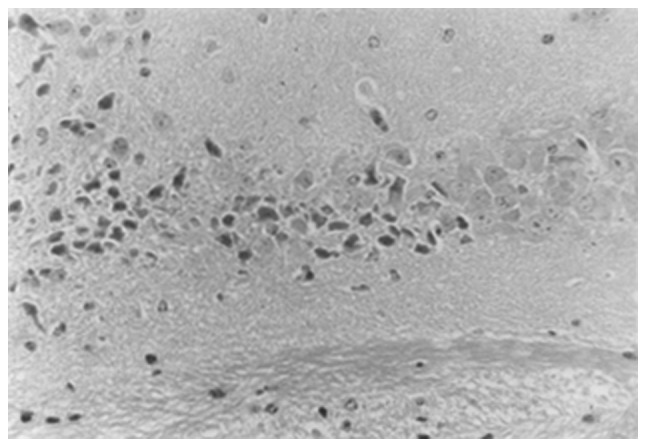
Model 30 day group: significantly reduced number of neurons compared with the model 10 day group, which are loosely arranged and irregular, a large number of heavily stained, irregularly shaped glial cells, and only a small number of normal nerve cells (hematoxylin and eosin staining; Magnification, ×50.).

In the sham group, large numbers of hippocampal CA1 neurons were observed, which were tightly and neatly packed, with rounded nuclei, prominent nucleoli and uniform chromatin. The number of neurons 10 days and 30 days following surgery showed no morphological difference (Figs. 1 and 2). However, in the model group, 10 days following surgery, the hippocampal CA1 neurons were reduced in number and showed a disorganized arrangement. The nerve nuclei were deeply stained and pyknotic, with large irregularly shaped glial cells. The number of hippocampal CA1 neurons was significantly reduced after 30 days compared with the number at 10 days, with an irregular arrangement. In addition, an increase in the number of glial cells was observed, which were strongly stained and irregular in shape. Only a small number of normal neurons were observed (Figs. 3 and 4).

## Discussion

With life expectancy increasing every decade, dementia is a growing problem. Dementia has been a major cause of morbidity worldwide, and the total number of patients with dementia is 50 million, with 10 million in China; however, 35 million are thought to have not yet been diagnosed (5). Pharmacological treatments to delay the progression of cognitive impairments are modestly successful and novel therapies are required to improve the cognitive deficits associated with dementia (6).

Memory and cognitive dysfunction, including VaD, are usually caused by ischemic and hypoxic damage to the brain. Previous studies have found that the pathogenesis of VaD is due to delayed neuronal death in the hippocampal CA1 area, and repeated cerebral ischemia is an important cause of the development of VaD. In the present study, repeated cerebral IR combined with blood pressure reduction was performed to establish the mental retardation model of behavioral changes in mice. The learning and memory function of these model animals was severely impaired, which was demonstrated by the results from the step-down avoidance and water maze behavioral tests. In addition, the number of neurons in the hippocampal CA1 area of these mice was observed to be significantly reduced by light microscopy, indicating that the vascular dementia animal model was established.

At present, rat models are commonly used to investigate vascular dementia; however, there is a lack of unified and recognized methods for the establishment of rat models. A number of different methods have been reported to establish VaD cerebral ischemia models. i) Bilateral carotid artery ligation model (7): Rats subjected to bilateral common carotid artery occlusion or 2-vessel occlusion have been used as animal models of subcortical ischemic vascular dementia (8). The therapeutic potential of certain drugs has also been evaluated following their administration to rats with experimental VaD, established by permanent bilateral common carotid artery occlusion (BCCAO) (9). ii) In another study, 6-month old rats were exposed to a diet rich in saturated fats and sucrose, and chronic BCCAO or sham surgery was performed (6). iii) Chronically hypertensive (for example, stroke-prone spontaneously hypertensive rats) exhibit certain features of VaD (10). iv) Numerous studies regarding ischemic brain damage in gerbils have been reported; however, studies on neuronal damage associated with various durations of IR are limited (11). In one study, a VaD model was established in gerbils that had been subjected to a transient cerebral ischemia, caused by 5 or 10 min occlusion of the bilateral carotid arteries, and the changes in the electrophysiological properties of hippocampal CA1 neurons seen 10 days following the 10-min cerebral ischemia were found to contribute to the impairment of spatial learning of the gerbils observed at this time (12). Furthermore, it has been identified that AMPK is transiently phosphorylated following forebrain ischemia in this model (13). v) Cerebral artery occlusion model (14): The animals undergo transorbital occlusion of the middle cerebral artery. vi) In another study, intercellular astrocytic Ca^2+^ waves were established by photochemistry in a mouse model of familial Alzheimer’s disease (15). Animal models of multiple ischemic lesions due to intra-vascular emboli (in rodents, rabbits or primates) have been established for dementia (16). Rat models of dementia established by intracerebroventricular injection of streptozotocin, and inhibition of brain mitochondrial cytochrome oxidase by sodium azide (17). These models have a number of disadvantages, including transient reversible learning and memory dysfunction (18), a craniotomy, high trauma, high animal mortality and being difficult to carry out (19).

To overcome the shortcomings of the aforementioned methods for the establishment of an animal model of VaD, including technical complexity, surgical trauma, high mortality and high production costs, in the present study a mouse model of VaD was established. This mouse model of cerebral ischemia may be used in behavioral observations, studies for the evaluation of drug efficacy, and screening experiments, and provides a valuable basis for the investigation of VaD. The model was successfully established in the present study. This mouse model has a number of advantages, including a low cost, adequate sources that are easy to rear, high survival rates, a simple modeling process, high success rate, low mortality, good reproducibility and marked pathological changes. Therefore, this model is worthy of application, and provides a foundation for further studies of the pathogenesis and drug and clinical treatment of VaD.

The mouse genome is only 80% homologous to that of humans, and dementia is closely associated with the age of humans. Although this study was limited to VaD in scope, the blood pressure of the mice was not detected after modeling. The pathology results confirmed the presence of dementia-like symptoms; however, this does not guarantee that all the model forms of VaD were consistent and comparable. Therefore, further studies are required in order to improve the repeatability of the model.

## Figures and Tables

**Table I:** Learning performance in the step-down avoidance test.

Group	n	Response period (sec)	Number of errors	Electric shock time (sec)
Sham 10 days	10	5.20±4.39	0.76±0.42	0.90±0.64
Sham 30 days	10	4.70±3.30	0.80±0.79	0.65±0.31
Model 10 days	10	19.00±5.62^a^	2.60±0.70^b^	14.00±4.27^b^
Model 30 days	10	50.60±44.33^b^,^c^	3.00±1.05^b^	49.00±44.59^b^,^c^

a P<0.01,

b P<0.001, versus the sham group;

c P<0.001, versus the model 10 days group.

**Table II:** Memory performance in the step-down avoidance test.

Group	n	Incubation period (sec)	Number of errors	Electric shock time (sec)
Sham 10 days	10	230.31±98.20	0.38±0.23	0.35±0.47
Sham 30 days	10	227.10±105.20	0.40±0.52	0.29±0.33
Model 10 days	10	41.7±33.27^a^	2.90±0.88^a^	6.20±3.77^a^
Model 30 days	10	15.10±16.74^a^,^b^	3.20±0.79^a^	9.60±2.99^a^

a P<0.001, versus the sham group;

b P<0.01, versus the model 10 days group.

**Table III:** Learning performance in the water maze test.

Group	n	Entire swim time (sec)	Number of errors
Sham 10 days	10	93.20±42.80	5.88±2.18
Sham 30 days	10	90.12±34.69	6.10±3.21
Model 10 days	10	187.40±76.80^a^	20.90±8.86^a^
Model 30 days	10	270.70±38.79^b^,^d^	29.40±5.44^b^,^c^

a P<0.01,

b P<0.001, versus the sham group;

c P<0.05,

d P<0.01, versus the model 10 days group.

**Table IV:** Memory performance in the water maze test.

Group	n	Entire swim time (sec)	Number of errors
Sham 10 days	10	93.00±42.35	10.50±6.85
Sham 30 days	10	92.54±52.60	6.50±3.18
Model 10 days	10	228.80±65.61^a^	22.50±6.57^a^
Model 30 days	10	269.80±28.26^b^,^c^	33.10±6.77^b^,^c^

a P<0.01,

b P<0.001, versus the sham group;

c P<0.05 versus the model 10 days group.
